# Evaluating the Comparative Effectiveness of Two Diets in Pediatric Inflammatory Bowel Disease: A Study Protocol for a Series of N-of-1 Trials

**DOI:** 10.3390/healthcare7040129

**Published:** 2019-11-01

**Authors:** Heather C. Kaplan, Lisa Opipari-Arrigan, Christopher H. Schmid, Christine L. Schuler, Shehzad Saeed, Kimberly L. Braly, Jennifer C. Burgis, Kaylie Nguyen, Sheri Pilley, Julie Stone, Gisele Woodward, David L. Suskind

**Affiliations:** 1 Perinatal Institute and James M. Anderson Center for Health Systems Excellence, Cincinnati Children’s Hospital Medical Center, Cincinnati, OH 45229, USA; 2 Department of Pediatrics, University of Cincinnati College of Medicine, Cincinnati, OH 45229, USA; Lisa.Opipari@cchmc.org (L.O.-A.); christine.schuler@cchmc.org (C.L.S.); 3 Division of Behavioral Medicine and Clinical Psychology and James M. Anderson Center for Health Systems Excellence, Cincinnati Children’s Hospital Medical Center, Cincinnati, OH, USA; 4 Department of Biostatistics, Center for Evidence Synthesis in Health, Brown University School of Public Health, Providence, RI 02912, USA; christopher_schmid@brown.edu; 5 Division of Hospital Medicine, Cincinnati Children’s Hospital Medical Center, Cincinnati, OH 45229, USA; 6 Division of Pediatric Gastroenterology, Dayton Children’s Hospital, Wright State University, Dayton, OH 45404, USA; SaeedS@childrensdayton.org; 7 Independent Consultant, Division of Pediatric Gastroenterology, Seattle Children’s Hospital, Seattle, WA 98105, USA; kim@kimbralynutrition.com; 8 Division of Pediatric Gastroenterology, University of California San Francisco, San Francisco, CA 94158, USA; Jennifer.burgis@ucsf.edu; 9 Division of Pediatric Gastroenterology, Lucile Packard Children’s Hospital, Stanford University, Palo Alto, CA 94304, USA; kanguyen@stanfordchildrens.org; 10 Parent Working Group, ImproveCareNow, Inc., Burlington, VT 05405, USAstone.juliem@gmail.com (J.S.); giselewoodward@yahoo.com (G.W.); 11 Division of Gastroenterology, Hepatology and Nutrition Seattle Children’s Hospital, University of Washington, Seattle, WA 98105, USA; david.suskind@seattlechildrens.org

**Keywords:** inflammatory bowel disease, Crohn’s disease, ulcerative colitis, specific carbohydrate diet, ImproveCareNow

## Abstract

Inflammatory bowel disease (IBD) affects 3 million children and adults in the US. Treatment involves medications with considerable risk profiles. Dietary modification, such as the specific carbohydrate diet (SCD), may be helpful in treating IBD, but there is insufficient evidence of its effectiveness. N-of-1 trials are ideal for addressing this important research question. The Personalized Research on Diet in Ulcerative Colitis and Crohn’s Disease (PRODUCE) study employs a series of 50 individual N-of-1 trials that compare the SCD to a modified SCD. Treatment periods are assigned in blocks of two, with each patient completing two balanced treatment blocks. Patients are randomized to start with the SCD or modified SCD and alternate between conditions for four eight-week periods. A mobile app guides collecting and viewing data, transitioning diets, and reviewing personal results. Primary outcomes include patient reported outcomes (PROs) of stool frequency, stool consistency, pain interference, and gastrointestinal (GI) symptom severity. We examine changes in inflammation via fecal calprotectin. Participants will receive a personalized answer regarding comparative effectiveness between the SCD and a less restrictive diet option (modified SCD), as well as compared to their baseline diet. We will aggregate the results of completed N-of-1 trials across patients to estimate population level comparative effectiveness of these treatments and the effectiveness of each diet.

## 1. Introduction

Inflammatory bowel disease (IBD) is a condition that affects 3 million adults and nearly 100,000 children across the United States [1,2]. The morbidity associated with childhood IBD, including Crohn’s disease and ulcerative colitis (UC), is substantial. Patients with IBD often experience symptoms such as abdominal pain, diarrhea and bloody stools, delayed growth, as well as impaired quality of life with missed school, hospitalizations, and in some cases, surgery [3,4]. Although effective treatments exist for IBD, patients often report residual symptoms and disease flares that impact their health and well-being [5,6,7]. Patients unable to achieve disease control with typical therapies, and those that are apprehensive regarding immunosuppressive medications and biologics, often seek alternative therapies, such as diet modification, as primary or adjunctive treatments [8].

Aside from the use of exclusive enteral nutrition, evidence for the role of diets in managing IBD is limited [9]. Preliminary data regarding the specific carbohydrate diet (SCD) suggest that it may result in clinical benefits and improvements in inflammatory biomarkers [10]. The SCD focuses on nutrient dense whole foods, including fruits, vegetables, nuts, meats, and lactose-free dairy, while excluding grains and certain complex sugars [11]. Several small retrospective and prospective studies suggest improvement in clinical symptoms and inflammatory markers within two–three months of initiating the SCD, that are maintained through 52 week follow-up [10,12]. Despite the promise of the SCD, there is no evidence of its effectiveness compared to unrestricted diets from controlled, large scale, multi-center studies. There is also little evidence of the effectiveness of the SCD compared to other less restrictive diets, which is important given the burden associated with exclusion diets. If the SCD can effectively induce and maintain remission, then patients will have a viable additional therapy to current standard medical treatments.

N-of-1 clinical trials are a useful design for studying dietary therapy, such as the SCD, in children with IBD. N-of-1 trials may be particularly informative in this case because considerable heterogeneity in dietary treatment effects in IBD is expected, given the variation in food metabolism, microbiota, and disease pathology across patients. This makes a study design that provides personalized results very appealing. Beyond individual results, aggregating the data across the series of N-of-1 trials to obtain estimates of population effectiveness of dietary therapy will contribute to the evidence base for the role of the SCD in IBD. Here, we outline our approach to performing a series of N-of-1 clinical trials to assess the effects of dietary modification on symptoms and inflammation in children with IBD.

## 2. Materials and Methods

### 2.1. Study Design

The Personalized Research on Diet in Ulcerative Colitis and Crohn’s Disease (PRODUCE) study will involve a series of individual N-of-1 trials that compare the SCD to a modified version of the SCD using a collaborative approach between patients, parents, and their clinical team (clinical trials.gov #NCT03301311). Each N-of-1 trial will utilize a double cross-over design (e.g., ABAB or BABA). Patients will enter the N-of-1 trial on an unrestricted baseline diet and will maintain that diet for one to two weeks before starting their initial treatment period. Length of time in the baseline period will be determined by the family and medical team based on the patient’s health status and family readiness to begin the new diet. While a longer baseline period would be preferable for data analysis, this was not deemed feasible or safe for pediatric patients who were in need of treatment. The study was approved by the Cincinnati Children’s Hospital Medical Center Institutional Review Board (IRB 2017-0683).

**Figure 1:**
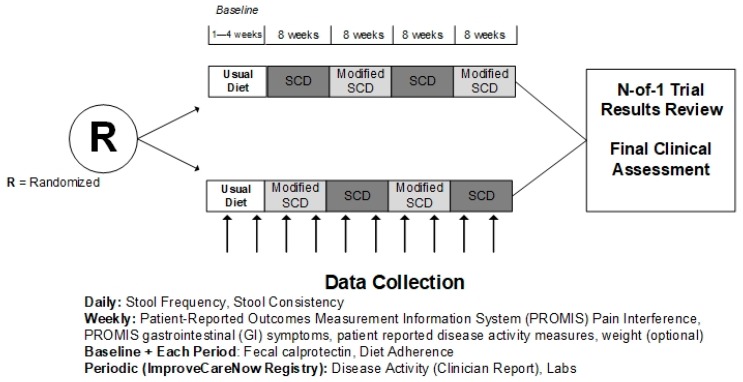
Study design.

Patients will be randomized to begin with either the SCD or the modified SCD. By randomizing the initial diet, we will determine whether effects differ based on the diet a patient starts with. Currently, there is uncertainty regarding whether starting on the full SCD diet before liberalizing is important. Patients will be randomized with a 1:1 allocation ratio using a centralized, stratified, block randomization approach. We will stratify within sites and by disease type (UC/indeterminate colitis (IC) or CD). To maintain allocation concealment, we will randomize patients in blocks of size 2 or 4 within each stratum, with block size chosen randomly. We will assign treatment periods in blocks of two, each including eight weeks on diet ‘A’, followed by eight weeks on diet ‘B’, with order of treatments dependent on randomization assignment. This design ensures two balanced blocks of time (e.g., AB) for each patient. In total, there will be four eight-week periods that alternate between the SCD and modified SCD (Figure 1). The eight-week treatment period duration was selected based on preliminary studies indicating that symptoms improve in approximately one month and markers of inflammation within two months.

### 2.2. Study Population

We plan to enroll 50 patient participants at 21 ImproveCareNow (ICN) Centers [13] between May 2018 and November 2019. Inclusion and exclusion criteria are listed in Table 1. Established in 2007, the ICN network is a collaborative community where clinicians, researchers, parents, and patients are empowered to learn and continuously improve, to bring about improved care and better health for children and adolescents with IBD. The 105 participating centers collect standardized data during all clinical visits which is entered into a registry, monitor individual and overall performance, compare outcomes, share the best evidence and tools, and participate in research.

### 2.3. Interventions and Comparators

We will define a patient’s usual diet, or baseline condition, as their typical, non-SCD diet. Often children with IBD have some dietary restrictions, such as seeds, nuts, or gluten, however baseline diets do not typically have broad restrictions, such as the exclusion of grains, sugars, or dairy. Baseline diets may include oral liquid supplements.

Each participant’s first diet period (whether the patient is randomized to the SCD or modified SCD) will begin with a two to three day transition from the usual, baseline, diet to their initial treatment diet. The SCD diet will be defined according to previously published guidelines [11]. Foods permitted on this diet include: meat/fish/poultry, eggs, some legumes (e.g., lentils and split peas are permitted, chickpeas and soybeans are not), 24-h fermented yogurt, non-starchy vegetables, ripe fruit, nuts/seeds, honey, and nut flours (e.g., almond flour or coconut flour). Grains, milk products aside from 24-h fermented SCD yogurt and cheeses aged greater than 30 days, starchy vegetables, processed foods, good additives, and natural or artificial sweeteners, aside from honey, are not allowed [14].

The modified SCD (MSCD) is a more liberal version of the SCD. Patients on this diet, in addition to the permitted foods on the SCD, will also be permitted to have organic white or brown rice, oats, sweet potatoes, grade A maple syrup, 100% unsweetened cocoa powder (not Dutch processed), and 100% cacao nibs or cacao butter (no sugar added). We will set weekly minimum and maximum intake requirements for the additional foods on the MSCD to ensure there is sufficient distinction between the modified SCD and strict SCD.

### 2.4. Technology Platform Supporting N-Of-1 Trials

**Figure 2:**
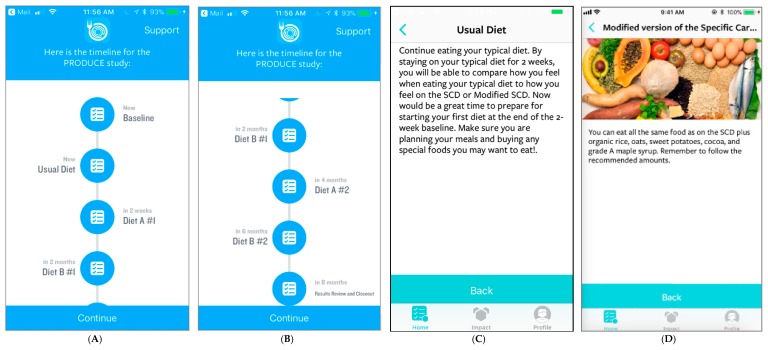
Screen shots of study timeline, including start (**A**) through completion (**B**) and information on usual diet (**C**) and the modified specific carbohydrate diet (**D**), as viewed through the participant mobile application.

**Figure 3:**
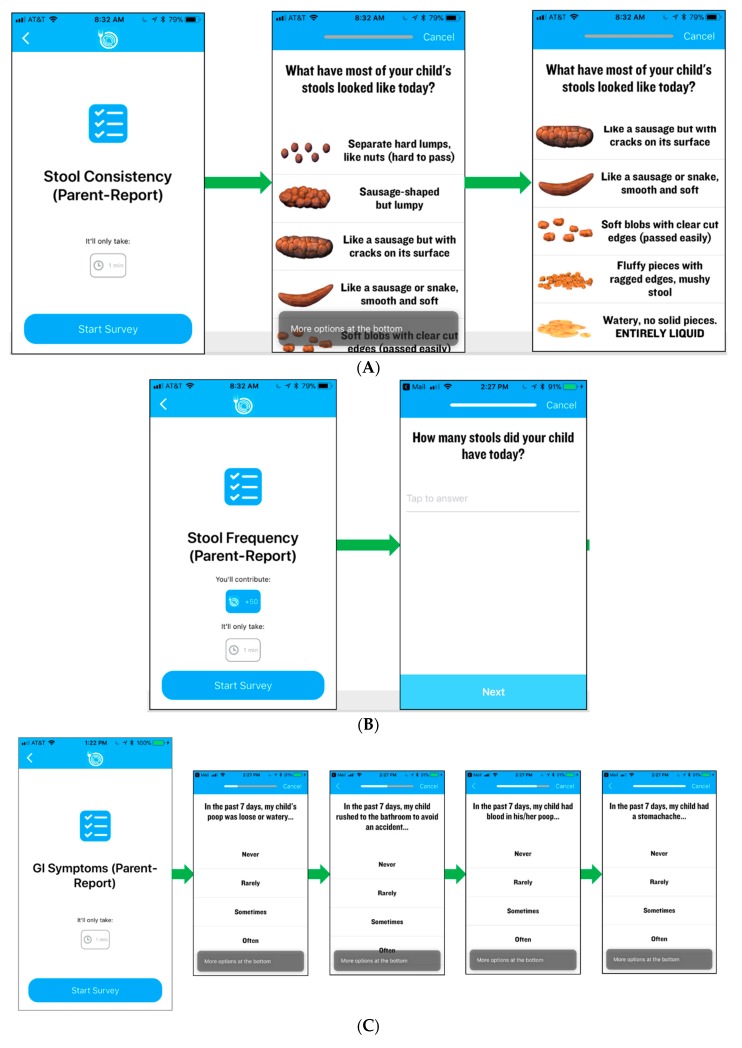
Screen shots of the participant facing mobile application data entry screens for (**A**) stool consistency, (**B**) stool frequency, and (**C**) Gastrointestinal (GI) symptoms.

**Figure 4:**
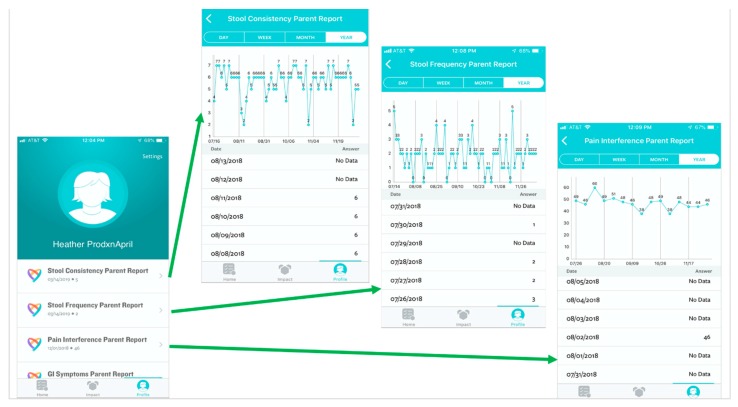
Screen shots displaying real-time, patient-level data as viewed through the participant mobile application.

We will tailor the Eureka digital research platform to support the execution of this study. Eureka is a digital platform, sponsored by the National Institute of Health, that was designed to facilitate mobile and internet-based research by allowing researchers to customize the existing platform architecture to meet the needs of their specific study. This Health Insurance Portability and Accountability Act (HIPAA) compliant platform provides a participant-facing mobile app designed to guide participants through the duration of the study (Figure 2). Additionally, there is a web-based investigator portal and a secure “back end” for data storage and analyses [15]. Participants will input outcome data (Figure 3), track treatment periods, and be able to review collected data in real time (Figure 4) via the app. At the time of study completion participants and clinicians will use the web-based platform to jointly view final results, including graphics and probabilistic assessments (Supplementary Materials Figure S1).

### 2.5. Study Procedures

#### 2.5.1. Baseline Evaluation and Training

Participants will have a routine clinical assessment at a standard of care visit prior to entering this study, where providers will assess disease activity and measures of inflammation, which may include fecal calprotectin, fecal lactoferrin, C-reactive protein (CRP), and/or erythrocyte sedimentation rate (ESR). At their enrolment visit, the patient will be randomized to their starting diet, and a study dietitian will counsel each patient individually on how to implement the diet and provide print and web-based [16] resources for future reference. Patients and parents will download the Eureka app to their smartphone and will be trained in the use of the app to track symptoms and monitor progress throughout the study. Participants will complete their first symptom surveys as well as a baseline demographic survey prior to leaving the baseline evaluation. In addition, participants will be instructed to complete and return a three-day diet history to assess baseline dietary intake and a stool sample to test for fecal calprotectin.

#### 2.5.2. Follow up Contact

In accordance with standard care practices, each patient will be evaluated by a provider and/or dietitian 2 ± 1 week(s) after starting each of the two study diets. The goals of these evaluations are to assess participant weight and nutritional intake and to provide diet education, sample meal plans, and resources for implementing the diets.

A provider follow-up visit will occur 8 ± 2 weeks from initiation of the first diet period, at which time weight, height, medication, laboratory and clinical disease activity data will be collected. Additional provider follow-up visits will be determined by the typical care practices at each site and patient care needs.

Each patient will complete a three-day diet diary around the mid-point of each respective treatment period. Diet diaries will be analyzed to provide nutritional analysis using Food Processor^®^ Nutrition and Fitness Software (11.6), © (2019) (ESHA Research, Inc. Salem, OR, USA) on the participants’ nutritional composition will be provided back to the dietitians. Dietitians will follow up with patients by phone once during each treatment period to review results of the diet diary and nutritional analysis (if available) and to discuss the adequacy of the diet and recommend any necessary adjustments or vitamin supplementation. They will also systematically assess adherence to the diet.

#### 2.5.3. Symptom Tracking

Participants will track symptoms and self-reported measures of disease activity (see outcome measures) via the Eureka mobile app (Figure 3). Parents will track information for patients younger than 14 years. Patients 14–18 years may also track their information along with their parent. Both clinicians and participants will have access to visualize the data in real-time via the mobile app (participant), as shown in Figure 4, or web portal (clinician).

#### 2.5.4. Stool Collection

We will collect stool samples at the baseline visit, as well as at the end of each respective treatment period, for fecal calprotectin and biobanking. Participants will not be able to advance to their next treatment block until a stool sample is submitted. Fecal calprotectin results will be made available to the clinicians as the results are available.

#### 2.5.5. Study Completion and N-Of 1 Results Review

Each participant will follow-up with their physician or provider within four weeks of completing their final treatment period for a standard medical visit and to review the results of the N-of-1 trial via the Eureka platform (Supplementary Materials Figure S1). Participants will complete one final questionnaire regarding their experience with the N-of-1 trial. 

### 2.6. Outcome Measures

#### 2.6.1. Primary Outcomes

The primary outcomes of this study include a suite of measures of IBD symptoms and disease activity plus fecal calprotectin as a measure of intestinal inflammation, as outlined in Supplementary Materials Table S1. Symptom and disease activity measures will be collected via the mobile application, which incorporates push notifications to prompt participants to enter information. Parents will respond using proxy surveys for participants under age 14. Symptom measures of stool frequency and stool consistency are assessed daily. Stool consistency is reported using the Bristol scale [17]. Measures of pain interference and gastrointestinal (GI) symptoms from the Patient-Reported Outcomes Measurement Information System (PROMIS) [18] item-bank will be assessed weekly. In addition, participants will self-report their disease activity weekly using the Short Crohn’s Disease Activity Index (sCDAI) [19] or self-reported version of the Pediatric Ulcerative Colitis Activity Index (PUCAI) [20]. Participants will mail in stool samples once per period to a centralized site using necessary packaging procedures to maintain sample integrity. Samples will be analyzed for fecal calprotectin at a single laboratory.

#### 2.6.2. Secondary Outcomes

We will follow multiple secondary outcomes through this study, obtained through the ICN registry. The ICN registry is a standardized clinical registry that collects IBD-specific data about processes and outcomes of care. For the purposes of this study, we will use data captured in the ICN registry that are electronically or manually imported from the electronic health records (EHRs) at each respective study site. Specifically, we will use data from the ICN registry on demographics, baseline characteristics, medications, and changes in disease activity/inflammation and weight during the course of the N-of-1 trial. Secondary outcomes will include growth (weight and height z-scores), clinician-reported disease activity as assessed by the short Pediatric Crohn’s Disease Activity index (sPCDAI) [21] and PUCAI [20], and laboratory markers of inflammation, including CRP, albumin, hematocrit, and ESR (Supplementary Materials Table S1). These outcomes are assessed at a frequency determined by the provider and local site practice and are not dictated by the study protocol.

#### 2.6.3. Safety Outcomes

To ensure patient safety through these N-of-1 trials, we will monitor for adverse events as reported to sites during clinical encounters or study activities. We will report any harmful incidences that are temporally associated with the subjects’ participation in the research, including worsening symptoms, emergency visits, hospitalizations, and any unanticipated surgical procedures. Additionally, we will monitor patients for weight loss via weight checks during clinical visits and if participants choose to weigh themselves on a home scale weekly and record this information in the Eureka app. In addition, a data safety and monitoring board (DSMB) will meet annually to assess participant safety.

## 3. Results and Analysis

### 3.1. N-Of-1 Individual Trials

At the end of each person’s N-of-1 trial, we will compare the difference in average symptom response to the two treatments (SCD and modified SCD) and on each treatment compared to the baseline. We will report results to patients regarding stool frequency, irregular stool consistency, pain interference, GI symptoms, and fecal calprotectin numerically and graphically. As we expect that effects on symptoms from a previous diet will wash out after one week on a new diet, we will discard the first seven days of data, including the first weekly measure during each treatment period. We will present bar graphs of the mean responses in the baseline, SCD, and modified SCD phases. For symptom measures, we will also present posterior means from a Bayesian analysis using a noninformative prior for the means and variance of the difference in responses across comparisons (e.g., comparing baseline versus SCD, baseline versus modified SCD, and SCD versus modified SCD). Finally, we will present to each patient a posterior probability of an improved or worsened response to each respective diet. For binary and count outcomes, we will report the probability that the log relative risk is no more than one standard deviation from zero for comparisons of the more complex treatment as compared to the simpler one (e.g., SCD versus modified SCD, SCD versus baseline diet, or modified SCD versus baseline diet). For PROMIS measures, the threshold is a mean difference of 2.9 points in either direction [22]. Models will incorporate the appropriate distributions for different outcome scales and link functions connecting the expected outcome to predictors for continuous, binary, count, and ordinal variables. We will not include autocorrelations in models, given the limited data collection (weekly intervals) and low suspicion for large correlation between measurements. We will also have limited measurements to estimate complex nonlinear effects, and do not expect these to be sizable, so we will not adjust for trend. However, we will investigate correlated error and trend models retrospectively in sensitivity analyses.

Our open-source R software will incorporate data from the Eureka platform, create the Bayesian model, and identify intelligent starting values for modeling. These starting values will be fed into open-source Just Another Gibbs Sampler (JAGS) software through R using the Rjags package to generate the Markov chain Monte Carlo simulations of the joint posterior distribution [23]. Model results will be returned back to the Eureka platform to be displayed for each study participant. We will provide individualized results to participants completing at least one paired treatment period with enough data in both treatment periods for valid statistical analysis. With regard to fecal calprotectin, given the limited number of measurements (five), we will provide graphic depictions of raw results (or average) in the baseline, SCD, and modified SCD periods. For children younger than 14 years, we will return results based on the parent data only. If children are 14 or older, and data are available from both child and parent, we will use that data stream which is most complete (either child or parent, but not mixed).

### 3.2. Meta-Analysis of N-Of-1 Trials

We will aggregate results from all individual N-of-1 trials and use Bayesian multilevel generalized linear mixed models with non-informative priors to estimate posterior distributions of within- and between-patient treatment effect sizes, as well as the average treatment effect in the population of patients studied. The within-patient estimates describe an improved estimate of each patient’s true effect, assuming exchangeability across patients. We will compare the multilevel patient estimates with those from the patient’s data alone in order to determine how much the effects change and how much additional precision these estimates gain. We will also fit models, including treatment effects, autocorrelated errors, carry over, and time trends (e.g., cubic splines). Additionally, we will conduct sensitivity analyses, investigating several different carryover models to determine whether an effect exists, and if so, how long it might last. The primary model will assume that carryover will be less than one week and will include a one-week analytic washout period. We will also investigate other analytic washouts of different lengths, however, to determine if one week is sufficient for the washout or whether a shorter period might be adequate.

We will use the parent data only for these analyses if children are younger than 14 years. In cases where both the child and parent are tracking, we will analyze the data from different sources as follows: (1) analyze all parent data; (2) analyze all child data; (3) analyze a single stream of data that uses the data source that is most complete; and (4) combine parent and child data by using whichever data item is available on a given day and using the average if both parent and child report.

### 3.3. Secondary Outcomes Collected at Clinic Visits

We will use Bayesian multilevel models for the meta-analysis of the N-of-1 outcomes, including disease activity measures (PUCAI [20] and sPCDAI [21] scores from the ICN registry), measures of inflammation (ESR, CRP, Hematocrit, and Albumin) and growth (weight for age z-score). We will perform these analyses using data collected through the ICN registry at clinical assessments throughout the duration of the 34-week study to examine the effectiveness of the SCD and modified SCD across, but not within, individuals. All clinical data in the ICN registry used for analyses will be linked electronically to the N-of-1 treatment periods for each respective patient. Assessments in the first week of a given intervention period will be assigned to the previous period, as the timing of the visit is likely to correspond to the initial visit before the transition from one period to another.

### 3.4. Heterogeneity of Treatment Effects

We will examine the heterogeneity of treatment effects between patients by including interactions for treatment with site, disease, and between-patient modifiers, including demographics, child and parent well-being, expectations of benefit, ICN center, diagnosis (UC/IC or CD), anatomic location of disease at enrollment, time since diagnosis, medications, and diet adherence.

### 3.5. Sample Size Determination

Our primary goal is to determine the effectiveness of each respective diet at the individual level. Therefore, it is not necessary to protect against a false-positive decision as in standard hypothesis tests used in clinical trials; thus, sample size calculations are not recommended for individual N-of-1 trials [24]. However, when estimating the population average effects of the SCD versus modified SCD on improving symptoms, a total of 50 patients will be needed to participate in the two-treatment crossover study. We will have 90% power with a two-sided 5% alpha to detect a mean difference of 3 points between groups [22]. This power calculation uses the weekly PROMIS pain interference T-score measure (standardized mean of 50 and standard deviation of 10) and incorporates aspects of the study design, including planned crossovers, washout periods, and autocorrelation from repeated measures. In particular, it assumes that the autocorrelation is no more than 0.5 or the standard deviation of the treatment difference is not greater than 5.

## 4. Discussion

We anticipate that our proposed use of N-of-1 trial methodology will demonstrate the potential of this trial design in children with IBD. Using the N-of-1 trial approach, we will generate individualized results and population-based estimates establishing how dietary interventions, specifically the SCD, may improve symptoms and reduce inflammation in children with IBD. Gathering data efficiently and rapidly providing individualized results will empower patients and families to make informed decisions about diet-related management of IBD. In addition, it will provide greater insight into the suspected heterogeneity in treatment effects in this disease process. Simultaneously, aggregate results will inform the collective knowledge about the effects of the SCD and modified SCD in children with IBD. At the time of manuscript submission, 33 patients have been enrolled and 9 have completed at least one treatment pair (4 completed the entire N-of-1 trial, 5 completed one treatment pair).

The importance of patients as collaborators, and the anticipated value of their investment in this work, cannot be overstated. This study will be embedded in the ICN network. The goal of ICN is to transform health and healthcare delivery for children and adolescents with IBD by supporting patients, families, clinicians, and researchers in working together in a learning health care system to accelerate discovery, innovation, and the application of new knowledge [13]. As such, ICN patients and parents are equal members of the research team and collaborate on the design and execution of the study. Furthermore, N-of-1 trials are a highly valuable research method in the learning network setting. They support patients’ and families’ desire to be equal partners in care and care improvement. We hypothesize that the strong engagement of patients and families in ICN and the patient-centeredness of the N-of-1 approach will be important in the success of this study.

Although N-of-1 trials have been conducted in pediatric populations, our proposed trial will be the first of its kind in children with IBD and is also novel with respect to recruiting patients from an existing learning health network. Prior N-of-1 trials involving children have been conducted to examine the effects of attention deficit hyperactivity disorder (ADHD) medications on behavior [25], assess behavioral interventions in children with anxiety and depression [26], investigate alternative therapies in children with cancer [27], and study hypertension in children [28]. Collectively, however, N-of-1 trial methodology is not frequently used in pediatric populations. Therefore, employing this methodology in the setting of IBD, which affects 3 million patients, and doing so in the context of a multi-center, nation-wide learning health network, is novel with regard to population and potential to scale. This work will demonstrate that conducting large N-of-1 trials in pediatric patients with a chronic condition such as IBD is not only possible but can be done realistically with sufficient recruitment to generate meaningful aggregate results.

Despite their promise and evidence of benefit, rigorous N-of-1 studies are rarely conducted [29,30,31,32,33]. This may be due, in part, to perceived barriers, such as lack of knowledge and familiarity with analytical methods, the inconvenience of tracking, and burdensome time demands [32,34]. Mobile technologies address many of these barriers and can be leveraged to conduct N-of-1 trials. We believe that the study-specific configuration of an existing scalable research platform, as was done for this study, will decrease the burden and increase the efficiency of conducting N-of-1 research. The Eureka mobile application and web portal are reasonably integrated into systems used in clinical care and patient and family lifestyles, which we believe will facilitate broader use of N-of-1 trials as a research method. In addition, the inclusion of parent and patient collaborators in the configuration of the Eureka application was used to ensure the patient experience with the application would be optimized. Maximizing the patient experience with this application is of particular importance in this study, in which patients and parents need to maintain engagement over the 34-week trial period.

While we feel that an N-of-1 approach is an optimal design for this study based on the suspected heterogeneity of treatment effects and the patient community’s desire for personalized answers to this question, there are a number of challenges in using an N-of-1 design for our particular research question. Given that our interventions will be diet-based, blinding patients will not be possible. We plan to mitigate this factor, at least in part, by including objective outcomes, such as markers of inflammation. Due to the time it takes to see impact from diet changes, the duration of the N-of-1 trials is long and 34 weeks of study intervention may contribute to waning patient engagement over time. The eight-week diet period may also mean that patients have ongoing exposure to a diet that yields an unfavorable clinical response, although patients may elect to terminate an intervention period early if that occurs. Although we customized the Eureka platform for use in these N-of-1 trials, and engaged families in application development, it may not effectively meet the needs of study patients and provide a satisfactory user experience in displaying results. Additionally, as we plan to follow multiple outcomes in this work, it may be more difficult to draw meaningful inferences from those results than it might be if we had selected a single outcome measure to follow. However, even with the range of outcomes selected for these studies, we are not capturing all of the key outcomes that could change in response to dietary intervention (e.g., we are not measuring missed school or parental work). As this is a pragmatic study and patients are responsible for implementation of the dietary interventions within their daily lives, there is likely to be variation in adherence to the dietary interventions. While adherence to study interventions is not a problem specific to N-of-1 trials, it is highly relevant to interpreting each individual’s results and also will require an examination of differential effects of the diets based on dietary adherence in the meta-analysis.

## 5. Conclusions

N-of-1 trials hold great promise for a variety of pediatric populations, especially those burdened with chronic health issues such as IBD. This methodology might be particularly useful when patient populations already exist that are invested in improving outcomes, such as those involved in learning networks or patient-powered research networks (PPRNs). Collaboration with patients and families in study design and implementation should be a priority for research teams, and insights from these groups may increase the success of N-of-1 trials in mainstream research. The prioritization of families as collaborators and advances in the science of N-of-1 trials may enhance the pace of achieving the goal of individualized and personalized medicine.

## Figures and Tables

**Table 1:** Inclusion and exclusion criteria.

Inclusion Criteria	Exclusion Criteria	
Diagnosis of CD or UC or IC Age 7–18 years Enrolled in the ICN registry Evidence of acute inflammation and/or elevated acute phase reactant (1.5 times the upper limit of normal for fecal calprotectin and lactoferrin, or 1.15 times the upper limit of normal for CRP and ESR) within eight weeks of study enrollment.	Complex or unstable IBD Current or past (nine months) history of abscess, fistula, structuring CD, or ostomy Severe disease activity Hospitalization or surgery planned within three months Ongoing active gastrointestinal infection Severe malnutrition Recent medication changes Other complicating medical issues Other serious medical conditions	Serious psychological or psychiatric conditions Pregnancy Tobacco, alcohol, illicit drug use Inability to complete the protocol Non-English speaking On SCD/modified SCD within eight weeks of the study On a vegan diet Lack of smart phone and data plan Participating in another interventional study

Abbreviations: Crohn’s Disease (CD), Ulcerative colitis (UC), Indeterminate Colitis (IC), ImproveCareNow (ICN), Specific Carbohydrate Diet (SCD).

## Supplementary Materials

The following are available online at https://www.mdpi.com/2227-9032/7/4/129/s1, Figure S1: Screen shot of final results as viewed through the participant facing mobile application, Table S1: Primary and secondary outcome measures.
