# Supplementary material for: Evaluating the Comparative Effectiveness of Two Diets in Pediatric Inflammatory Bowel Disease: A Study Protocol for a Series of N-of-1 Trials
Source: Healthcare (Basel). 2019 Nov 1;7(4):129. doi: 10.3390/healthcare7040129 (PMC6956096; doi:10.3390/healthcare7040129)

Stool Frequency (Daily) | Stool Consistency (Daily) | Pain Interference (Weekly) | GI Symptoms (Weekly) | Fecal Calprotectin | Summary (All symptoms)

Did my Stool Frequency improve?

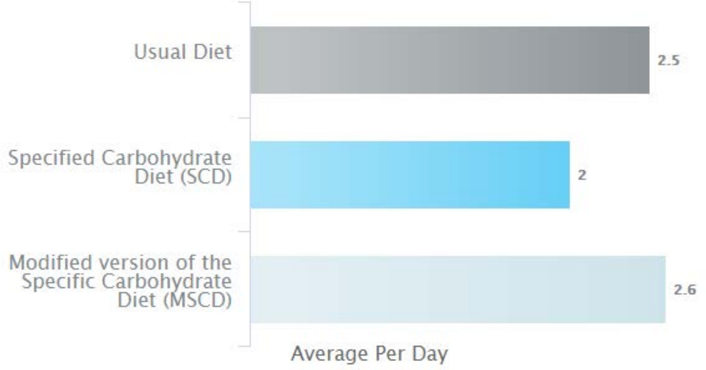

How the diets affected the number of stools you had:

- The SCD decreased your stool frequency by 0.5 stools per day compared to your usual diet.*
- The Modified SCD increased your stool frequency by 0.1 stools per day compared to your usual diet.*
- Compared to the Modified SCD, the SCD decreased your stool frequency by 0.6 stools per day.*

*There may be a difference between numbers on the bar graph (average scores) and numbers in the text (scores based on how your symptoms change over time and vary from day to day).

How Sure Can I Be About This Answer?

There are a number of factors that can influence how certain your results are. Results become less certain when you have fewer measurements, have not strictly followed the diets, and/or have made other changes at the same time that make it more difficult to isolate the effects of the diet.

Likelihood that SCD is Better than your Usual Diet for Stool Frequency

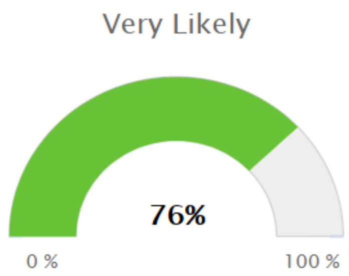

You have a 76% chance that you showed improvement in your Stool Frequency and a 4% chance your Stool Frequency got worse.

Likelihood that the Modified SCD is Better than your Usual Diet for Stool Frequency

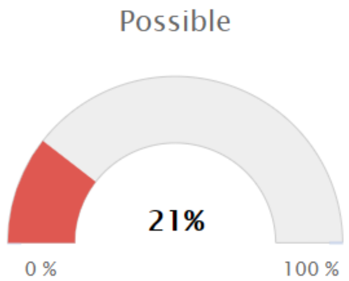

You have a 21% chance that you showed improvement in your Stool Frequency and a 40% chance your Stool Frequency got worse.

Likelihood that the SCD is Better than the Modified SCD for Stool Frequency

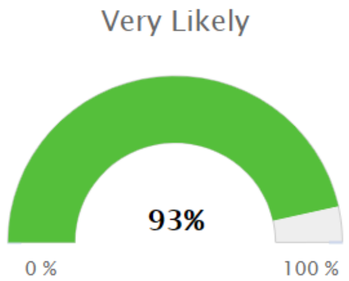

There is a 93% chance that your Stool Frequency was better on the SCD and a 1% chance that it was better on the Modified SCD.

Stool Frequency (Daily) | Stool Consistency (Daily) | Pain Interference (Weekly) | GI Symptoms (Weekly) | Fecal Calprotectin | Summary (All symptoms)

Did my Stool Consistency improve?

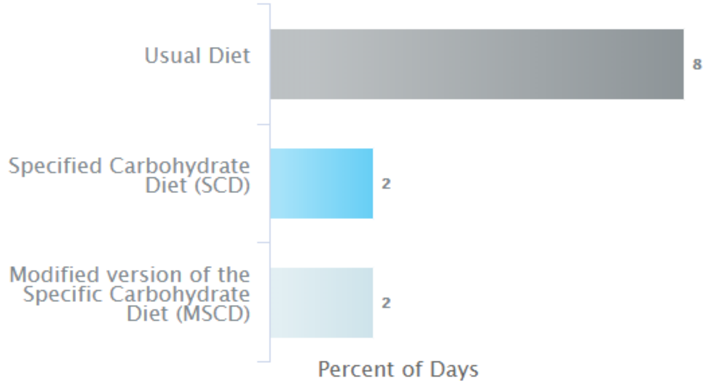

How the diets affected your stool consistency:

- The SCD decreased the percent of days you had irregular (loose or hard) stools by 5.0% compared to your usual diet.*
- The Modified SCD decreased the percent of days you had irregular (loose or hard) stools by 6.0% compared to your usual diet.*
- Compared to the Modified SCD, the SCD increased the percent of days you had irregular (loose or hard) stools by 1.0%.*

*There may be a difference between numbers on the bar graph (average scores) and numbers in the text (scores based on how your symptoms change over time and vary from day to day).

How Sure Can I Be About This Answer?

There are a number of factors that can influence how certain your results are. Results become less certain when you have fewer measurements, have not strictly followed the diets, and/or have made other changes at the same time that make it more difficult to isolate the effects of the diet.

Likelihood that SCD is Better than your Usual Diet for Stool Consistency

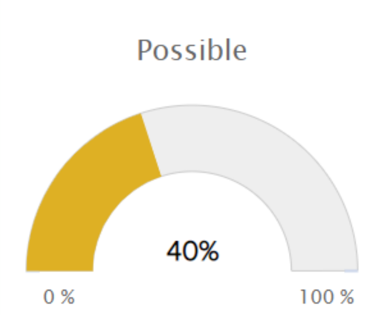

You have a 40% chance that you showed improvement in your Stool consistency and a 8% chance your Stool Consistency got worse.

Likelihood that the Modified SCD is Better than your Usual Diet for Stool Consistency

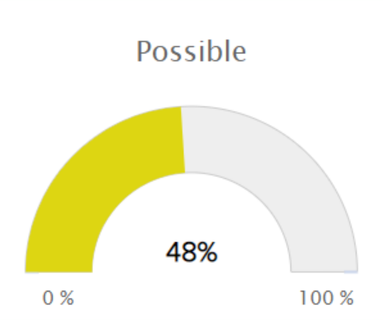

You have a 48% chance that you showed improvement in your Stool consistency and a 6% chance your Stool consistency got worse.

Likelihood that the SCD is Better than the Modified SCD for Stool Consistency

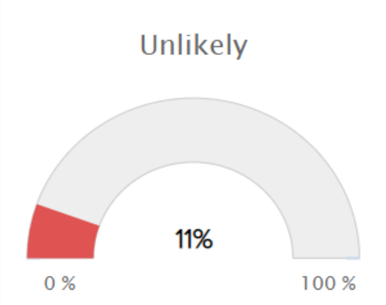

There is a 11% chance that your Stool consistency was better on the SCD and a 27% chance that it was better on the Modified SCD.

Stool Frequency (Daily) | Stool Consistency (Daily) | Pain Interference (Weekly) | GI Symptoms (Weekly) | Fecal Calprotectin | Summary (All symptoms)

Did my Pain Interference improve?

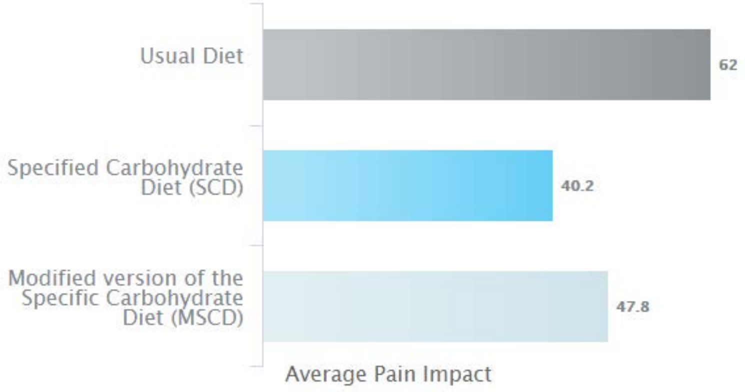

How the diets affected how much pain impacted your daily activities:

- The SCD decreased how much pain impacted your daily activities by 15.1 points** compared to your usual diet.*
- The Modified SCD decreased how much pain impacted your daily activities by 7.5 points** compared to your usual diet.*
- Compared to the Modified SCD, the SCD decreased how much pain impacted your daily activities by 7.6 points.* **

*There may be a difference between numbers on the bar graph (average scores) and numbers in the text (scores based on how your symptoms change over time and vary from day to day).

**A change of at least 3 points is generally considered meaningful

How Sure Can I Be About This Answer?

There are a number of factors that can influence how certain your results are. Results become less certain when you have fewer measurements, have not strictly followed the diets, and/or have made other changes at the same time that make it more difficult to isolate the effects of the diet.

Likelihood that SCD is Better than your Usual Diet for Pain Interference

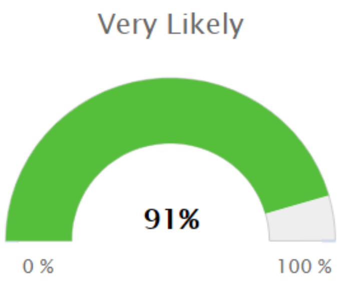

You have a 91% chance that you showed improvement in your Pain Interference and a 3% chance your Pain Interference got worse.

Likelihood that the Modified SCD is Better than your Usual Diet for Pain Interference

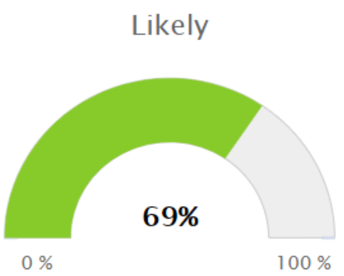

You have a 69% chance that you showed improvement in your Pain Interference and a 15% chance your Pain Interference got worse.

Likelihood that the SCD is Better than the Modified SCD for Pain Interference

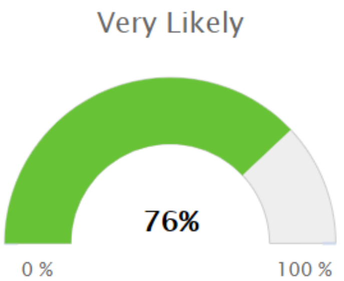

There is a 76% chance that your Pain Interference was better on the SCD and a 6% chance that it was better on the Modified SCD.

Did my GI Symptoms improve?

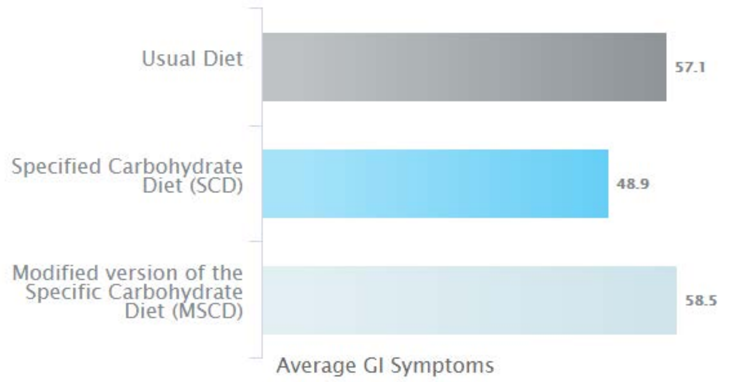

How the diets affected your GI symptoms:

The GI symptom measure includes an assessment of stomach pain, loose stools, the urgency to rush to the bathroom, and bloody stools.

- The SCD decreased your GI symptoms by 6.5 points** compared to your usual diet.*
- The Modified SCD increased your GI symptoms by 3.0 points** compared to your usual diet.*
- Compared to the Modified SCD, the SCD decreased your GI symptoms by 9.5 points.* **

*There may be a difference between numbers on the bar graph (average scores) and numbers in the text (scores based on how your symptoms change over time and vary from day to day).

**A change of at least 3 points is generally considered meaningful

How Sure Can I Be About This Answer?

There are a number of factors that can influence how certain your results are. Results become less certain when you have fewer measurements, have not strictly followed the diets, and/or have made other changes at the same time that make it more difficult to isolate the effects of the diet.

Likelihood that SCD is Better than your Usual Diet for GI Symptoms

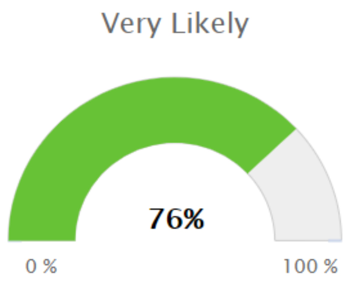

You have a 76% chance that you showed improvement in your GI Symptoms and a 4% chance your GI Symptoms got worse.

Likelihood that the Modified SCD is Better than your Usual Diet for GI Symptoms

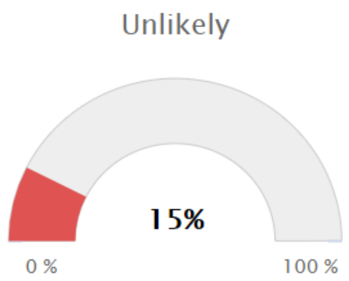

You have a 15% chance that you showed improvement in your GI Symptoms and a 50% chance your GI Symptoms got worse.

Likelihood that the SCD is Better than the Modified SCD for GI Symptoms

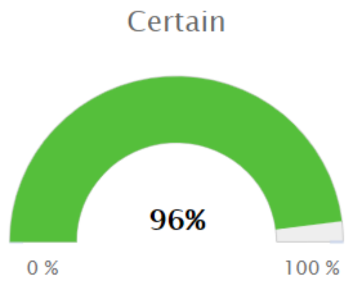

There is a 96% chance that your GI Symptoms was better on the SCD and a 1% chance that it was better on the Modified SCD.

Stool Frequency (Daily) | Stool Consistency (Daily) | Pain Interference (Weekly) | GI Symptoms (Weekly) | Fecal Calprotectin | Summary (All symptoms)

Did my Fecal Calprotectin improve?

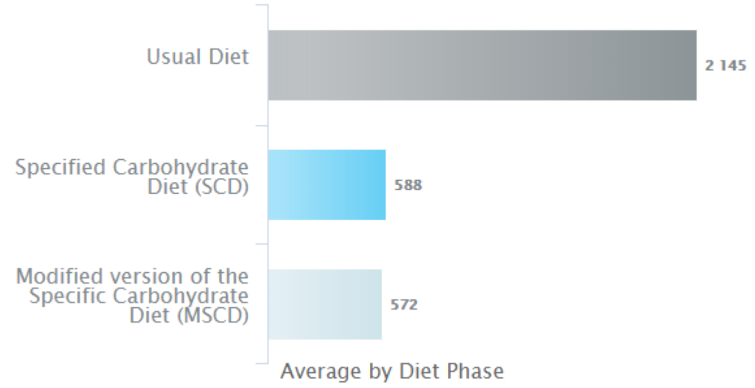

Fecal Calprotectin is a measure of inflammation in the bowel. Lower values suggest less inflammation. A normal Fecal Calprotectin is <=50 mcg/g .

Summary

What is the Chance that the SCD is better than the Modified SCD?

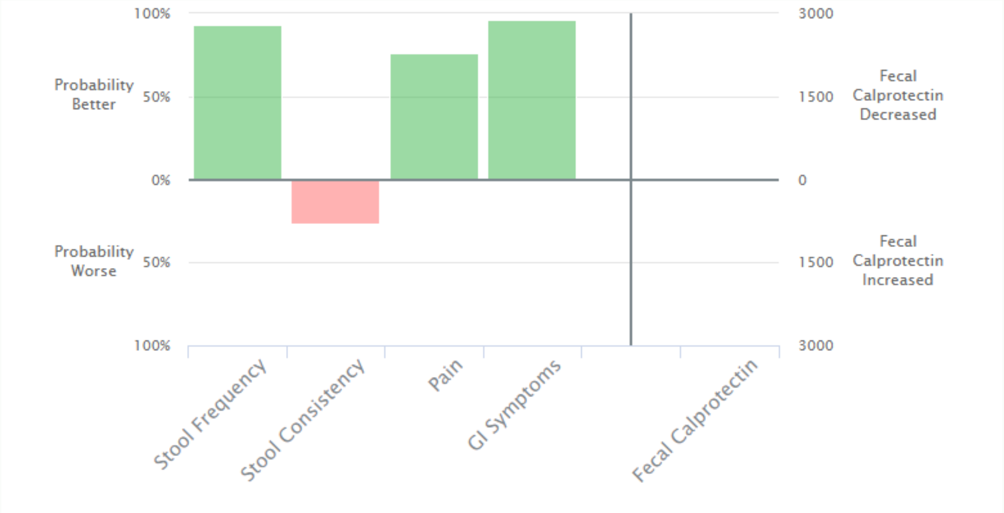

How does it all add up?

If your chance is high that you did better on the SCD versus the Modified SCD (for the symptoms that are most important to you), then you should weigh that information with impact of maintaining the SCD in your life.

If your chance that you did better on the SCD versus the Modified SCD is low, then you may be able to achieve the same benefit with the modified version of the diet.

Is the SCD Better than Your Usual Diet?

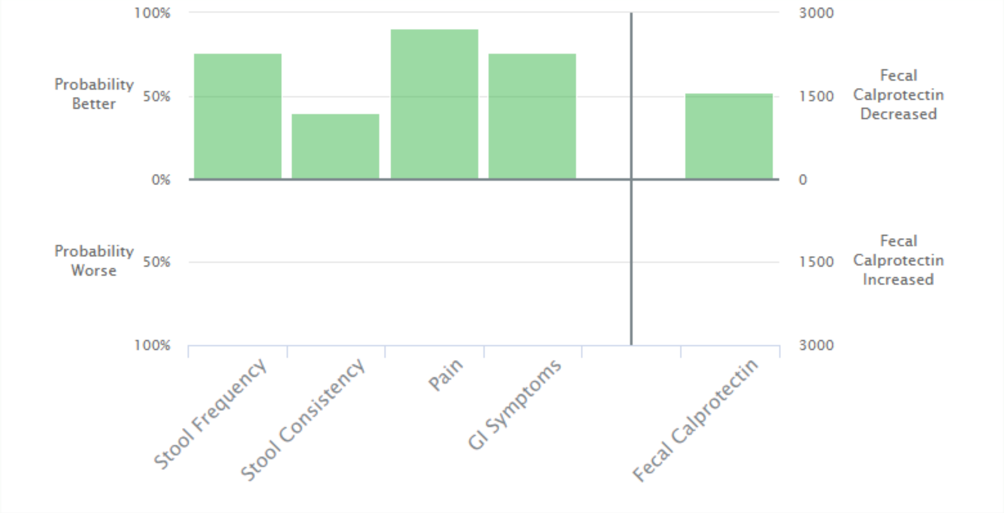

Is the Modified SCD Better than Your Usual Diet?

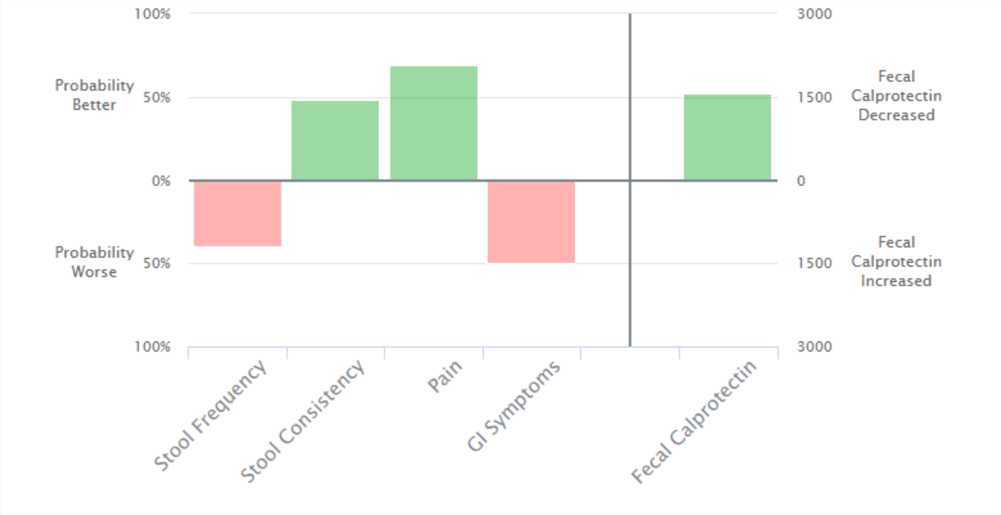

Supplement: Supplementary file 1 [file healthcare-07-00129-s001.zip › PRODUCE Protocol supplementary materials/Supplement Figure 1.PRODUCE_Final Results_Figure_de-identified.pdf]
