# Supplementary material for: Evaluating the Comparative Effectiveness of Two Diets in Pediatric Inflammatory Bowel Disease: A Study Protocol for a Series of N-of-1 Trials
Source: Healthcare (Basel). 2019 Nov 1;7(4):129. doi: 10.3390/healthcare7040129 (PMC6956096; doi:10.3390/healthcare7040129)
Supplement: Supplementary file 1 [file healthcare-07-00129-s001.zip › PRODUCE Protocol supplementary materials/Supplementary table.docx]

**Table S1.** Primary and secondary outcome measures.

| **Construct** | **Measure(s)** | **Categorization** | **Scoring/Scale** | **Timing** | **Source** |
| --- | --- | --- | --- | --- | --- |
| ***Primary Outcomes*** | | | | | |
| Stool Frequency | Single integer count | Integer | 0–infinity | Daily | Eureka app |
| Irregular Stool Consistency | Bristol Stool Scale  Ordinal (1–7) | Binary | Irregular Stool: 1 or 7  Regular Stool: 2–6 | Daily | Eureka app |
| Pain Interference | PROMIS® Pain Interference  (Child) | Continuous | Raw Score: 0–32  T-Score: 34–78 | Weekly | Eureka app |
|  | PROMIS® Pain Interference  (Parent Proxy) | Continuous | Raw Score: 0–32  T-Score: 38–78 | Weekly | Eureka app |
| IBD Severity | PROMIS® GI Symptoms  (Child) | Continuous | Raw Score: 4–20  T-Score: 39.8–79.2 | Weekly | Eureka app |
|  | PROMIS® GI Symptoms  (Parent Proxy) | Continuous | Raw Score: 4–20  T-Score: 39–80.3 | Weekly | Eureka app |
| Self-Report Disease Activity (UC/IC) † | Pediatric Ulcerative Colitis Activity Index (PUCAI) | Integer | 0–85 | Weekly | Eureka app |
| Self-Report Disease Activity (Crohn’s disease only) † | Short Crohn’s Disease Activity Index (sCDAI) | Integer | 44–infinity | Weekly | Eureka app |
| Intestinal Inflammation | Fecal Calprotectin | Continuous | 16–1800 mcg/gm | Weeks 0, 10, 18, 26, 34 | Lab |
| ***Secondary Outcomes*** | | | | | |
| Stool Consistency | Bristol Stool Scale | Integer | 1–7 | Daily | Eureka app |
| Disease Activity (UC/IC) | Clinician-reported Pediatric Ulcerative Colitis Activity Index (PUCAI) | Integer | 0–85 | Clinic Visits | ICN Registry |
| Disease Activity (Crohn’s disease only) | Clinician-reported  Short Pediatric Crohn’s Disease Activity Index (sPCDAI) | Integer | 0–90 | Clinic Visits | ICN Registry |
| Inflammatory & Disease Activity Markers | C-Reactive Protein | Continuous | 0.29-190 mg/dL | Clinic Visits | ICN Registry |
|  | Erythrocyte Sedimentation Rate | Continuous | 1–145 mm/hr | Clinic Visits | ICN Registry |
|  | Albumin | Continuous | 0.6–32 g/dL | Clinic Visits | ICN Registry |
|  | Hematocrit | Continuous | 10–50% | Clinic Visits | ICN Registry |
| Growth | Weight | Continuous | n/a | Clinic Visits | ICN Registry |

†These measures are primary outcomes, but will not be returned to participants with their individual N-of-1 results as they are similar to the PROMIS GI Symptoms measure.
